# FSH Beyond Fertility

**DOI:** 10.3389/fendo.2019.00136

**Published:** 2019-03-19

**Authors:** Daria Lizneva, Alina Rahimova, Se-Min Kim, Ihor Atabiekov, Seher Javaid, Bateel Alamoush, Charit Taneja, Ayesha Khan, Li Sun, Ricardo Azziz, Tony Yuen, Mone Zaidi

**Affiliations:** ^1^The Mount Sinai Bone Program, Department of Medicine, Icahn School of Medicine at Mount Sinai, New York, NY, United States; ^2^Academic Health and Hospital Affairs, State University of New York, Albany, NY, United States

**Keywords:** FSH, FSHR, obesity, osteoporosis, BMI, cardiovascular risk

## Abstract

The traditional view of follicle*-*stimulating hormone (FSH) as a reproductive hormone is changing. It has been shown that FSH receptors (FSHRs) are expressed in various extra-gonadal tissues and mediate the biological effects of FSH at those sites. Molecular, animal, epidemiologic, and clinical data suggest that elevated serum FSH may play a significant role in the evolution of bone loss and obesity, as well as contributing to cardiovascular and cancer risk. This review summarizes recent data on FSH action beyond reproduction.

## Introduction

Follicle*-*stimulating hormone (FSH) is long thought to exert its effects in gonadal tissues, mainly limited to Sertoli cells in testes and granulosa cells in ovaries (1). Recently, using methods such as RT-PCR, Sanger sequencing, immunohistochemistry, and competitive binding assays, FSH receptors (FSHRs) have been shown to be expressed in extragonadal tissues, including endothelium, monocytes, developing placenta, endometrium, malignant tissues, bone and fat (2–10).

Our group first demonstrated that by increasing bone resorption by osteoclasts, FSH regulates bone mass in mouse models (11). Moreover, we found that FSH exerts action on adipocytes. In particular, a novel FSH antibody blocks the action of FSH on FSHRs (10, 11), causing an increase in bone mass, a reduction of body fat and induction of beiging of white adipocytes (9). These findings are consistent with large epidemiologic data. Indeed, the Study of Women's Health Across the Nation (SWAN) has shown significant reductions in bone mineral density (BMD) and high resorption rates ~2–3 years prior to menopause, which was also associated with increased body weight and visceral adiposity (12, 13). It is important to note that these changes take place when serum FSH level is increasing and estrogen level remains normal (14). Emerging epidemiologic evidence also suggests a relationship between FSH and several cardiovascular risk factors such as coronary artery calcium deposition, carotid intima-media thickness, and the number of aortic plaques (15–17). In particular, FSH interacts with its receptor on monocytes, up-regulates RANK expression and promotes monocytic infiltration of atherosclerotic plaque (18).

With this new evidence, the view that FSH acts solely as a gonadal hormone has changed rapidly over the past decade. It also provides perspectives on new roles that FSH might have in the pathophysiology of certain diseases and how treatment approaches targeting FSH may open up new possibilities for prevention and treatment. For example, we showed that a FSH blocking antibody could prevent bone loss and visceral adiposity in various mouse models (10). These data provide the foundation for future human studies. Similarly, detection of vascular endothelial FSHR in various types of solid tumors and sarcomas has prompted a debate as to whether an anti-FSH antibody could serve as a treatment modality in future anti-cancer drugs (7, 19).

Here, we review the epidemiologic, molecular and animal data on FSH action in normal physiology and the pathophysiology of osteoporosis, obesity, cardiovascular disorders, and cancer.

### The Role of FSH in Reproduction

FSH, luteinizing hormone (LH), thyroid-stimulating hormone (TSH) and human chorionic gonadotropin (hCG) are all glycoprotein hormones, which share the same alpha subunit and differ in their beta polypeptide units, specific for each of aforementioned molecules (20). Pulsatile release of gonadotropin-releasing hormone (GnRH) from the hypothalamus stimulates the release of FSH and LH. Inhibin B and estradiol are the primary inhibitors of FSH secretion (21–23). Several other pituitary-regulatory proteins, such as activin and follistatin, have been implicated in FSH secretion and action (22). The activity of FSH is regulated in part by glycosylation.

FSH exerts its biological action via a G protein-coupled receptor, FSH receptor (FSHR). A stimulatory Gα_s_ protein initiates signal transduction via the cAMP/protein kinase A (PKA) pathway (1, 24). This cascade of events leads to the activation of cAMP regulatory element-binding protein (CREB) (24). In addition to CREB, cAMP-activated PKA activates several other factors such as p38 MAP kinases, p70-S6 kinase and phosphoinositide-3 kinase (PI3K), PKB/Akt and FOXO1 and regulates gene expression in target tissues (25, 26). According to recent data, the effect of FSHR activation is not limited to the classical pathway, but also produces its action through Gα_i_ (27), Gα_q_ (28), and via other molecules, including β-arrestins (29) and an adapter protein having pleckstrin homology and phosphotyrosine binding domains together with a leucine zipper motif (30). In this case, the signal transduction is accomplished though inositol trisphosphate (IP_3_), Akt and ERK1/2.

FSH plays a pivotal role in the development and regulation of both the male and female reproductive systems by acting on the FSHR which is predominantly expressed in granulosa and Sertoli cells (24). In females, FSH induces follicular growth and maturation, and contributes to LH-triggered ovulation and luteinization (31–33). In males, FSH regulates the mitotic proliferation of Sertoli cells, supports their growth and maturation and prompts the release of androgen-binding protein, which regulates the overall process of spermatogenesis (34). Moreover, in testis, endothelial FSHR mediates FSH transport across gonadal endothelial barrier (35). Below, we will discuss the role of FSH on bone, fat, cardiovascular system and cancer cells.

### Epidemiologic and Clinical Data Supporting FSH Action on Bone

Traditionally, bone loss in peri- and postmenopausal women has been attributed primarily to reduced estrogen production due to ovarian senescence. Estrogen replacement therapy has been considered a logical therapeutic choice in an attempt to slow postmenopausal bone loss and reduce fracture risk (36). However, FSH has been implicated in bone loss in reproductive and non-reproductive age women, as well as in women undergoing menopausal transition (37, 38).

While data from placebo-controlled randomized clinical trials is not available, the multi-center multi-ethnic cohort SWAN showed a compelling correlation between FSH action and bone loss during the menopausal transition. SWAN demonstrated that changes in bone turnover markers and bone mass density (BMD) in perimenopausal women undergoing menopausal transition were independent of serum estradiol, but were inversely related to changes in the FSH level. The levels of serum FSH over a 4-year time period predicted BMD reduction in these women (14, 39). Moreover, lower levels of bone loss in the lumbar spine during perimenopause were noted in women with higher estrogen-to-FSH ratio (40). All of these observations may suggest that bone loss during perimenopause is not solely dependent on estrogen, and may be due in part to FSH action on bone.

Epidemiological data from across the US, Europe, and China further substantiate findings from SWAN (41–46). The US NHANES III cohort study documented the relationship between serum FSH and femoral neck BMD among woman between the ages of 42 and 60 (41). Likewise, using univariate regression analyses, another US cross-sectional study confirmed the inverse relation of FSH to BMD in perimenopausal women (42). The Italian Bone Turnover Range of Normality (BONTURNO) study compared women undergoing menopausal transition, and showed significantly increased bone loss in the group with FSH>30 IU/L vs. age-matched controls, although both had regular menses (43). Yet another cross-sectional study conducted in Spain included 92 postmenopausal female participants and showed a positive correlation between serum FSH and C-terminal telopeptide of type I collagen (CTX) and serum osteocalcin, but no relation to estradiol. Several Chinese studies have reported a negative relationship between bone loss, bone turnover markers and serum FSH levels in perimenopausal women (45–47), with those in the highest quartile of serum FSH showing bone loss at a rate that was 1.3–2.3-fold higher than those in the lowest quartile (48).

The detrimental and deleterious effect of FSH on bone during a woman's reproductive years can be observed in instances of hypergonadotropic conditions. For example, lower lumbar spine bone density was reported in a hypergonadotropic amenorrheic group as compared to hypogonadotropic European patients under 40 years of age (49). Groups did not differ in estradiol or progesterone levels; however, in hypergonadotropic women, FSH levels had a negative relationship with lumbar spine BMD. Interestingly, females diagnosed with functional hypothalamic amenorrhea tend to develop less severe bone loss (50, 51).

Evidence from genetic studies further explores the function of FSHR in humans. In particular, women with an activating *FSHR*^*N*680*S*^ polymorphism have an increased risk of developing postmenopausal osteoporosis, independent of circulating levels of FSH and estrogens (52). Likewise, in a multicenter study of postmenopausal Spanish women two-gene combinations of wild type IVS4 or 3′UTR markers of *CYP19A1* with *FSHR* and *BMP15* genes yielded skeletal protection (53). Therefore, epidemiologic data derived from several cross-sectional and cohort studies, together with genetic association studies, suggest a detrimental effect of FSH on bone.

In contrast, a couple of clinical studies in humans using GnRH agonists failed to demonstrate any effect of FSH suppression on bone. For instance, FSH suppression with leuprolide acetate in a group of postmenopausal women has not being associated with any significant changes in bone resorption markers (54). In another study, eugonadal men receiving goserelin acetate combined with daily topical testosterone gel did not demonstrate any changes in serum N-terminal telopeptide, C-terminal telopeptide, and osteocalcin compared to control (55). However, both studies were relatively small and the duration of the intervention was short (approximately 4 months).

### Mechanistic Studies on FSH Action on Bone

In 2006, we were the first to observe the direct regulation of bone mass by FSH, which resulted mainly from osteoclastic bone resorption in rodents (11). Accumulating evidence now shows that FSH acts directly on bone via a specific shorter isoform of the FSHR (identified in humans), which then increases osteoclastogenesis and stimulates bone resorption (4, 11, 56–58). Studies failed to identify the expression of FSHRs on osteoclast lineage cells most likely used PCR primers designed to target the full-length gonadal FSHR (59, 60). FSH binding to the bone FSHR has subsequently been proven *in vivo* through the binding of fluorophore-tagged FSH to gonads and bone. A molar excess of unlabeled FSH displaced tagged FSH underscoring the specificity of FSH binding to bone (10, 61). The level of FSH glycosylation is important, as fully glycosylated (i.e., 24 kD) recombinant FSH isoform has a higher affinity to the bone FSHR, as compared to the partially glycosylated FSH molecule (i.e., 21 kD isoform), which is more active in gonads (62, 63).

FSH acts on FSHRs on osteoclasts, stimulating NFκB, MEK/Erk, and AKT pathways and, thus, promoting osteoclast formation, function and survival. The osteoclastic FSHR is coupled to Gαi_2_, so that its activation causes intracellular cAMP reductions, in contrast to the ovaries where the FSHR couples with a Gα_s_-protein and triggers an increase in cAMP. Blocking the aforementioned pathway or absence of Gαi_2_ leads to bone unresponsiveness to FSH (11). Stimulation of osteoclasts by FSH also occurs via an indirect pathway—the upregulation of receptor activator NFκB (RANK) increases the synthesis of interleukin-1β (IL-1β), interleukin-6 (IL-6) and tumor necrosis factor alpha (TNFα) proportionately to FSHR expression (64, 65). Moreover, FSH can interact with an immunoreceptor tyrosine-based activation motif (ITAM) adapter to enhance osteoclastogenesis (57).

*In vivo* FSH injection caused enhanced bone loss, whereas FSH inhibitor administration decreased bone resorption in ovariectomized rats (66, 67). Mice with an absent or deficient allele of FSHR or FSHβ had higher bone mass and diminished bone loss, which may be partially explained by high serum androgens (68). However, mice lacking aromatase, despite elevated androgen levels, still showed dramatic bone loss (69). Moreover, when FSH inhibitor was injected into male mice they also developed increased bone mass (9). To prevent confounding, generated by the opposite effects of FSH and estrogens on bone resorption, we developed a specific antibody to FSHβ (70, 71), which was shown to decrease osteoclastogenesis *in vitro* (10, 71), and decrease bone loss and stimulate bone formation *in vivo* (11, 70, 71). It is also known that FSH acts via the FSHR on mesenchymal stem cells to suppress their differentiation into osteoblasts (70).

### Epidemiologic and Clinical Data Supporting an Action of FSH on Body Composition

There is strong correlative evidence between high FSH and body fat in postmenopausal women. A Michigan sub-study of the SWAN, which included women undergoing menopausal transition, showed a positive relationship between fat mass and serum FSH. Participants with higher FSH had increased fat mass and waist circumference, even after adjusting for baseline measurements, and lower lean and skeletal muscle mass (72). In addition, the Oklahoma Postmenopausal Health Disparities Study, which included a large group of postmenopausal women, showed that the best predictors of waist-to-hip ratio were serum FSH, estradiol and body mass index (BMI) (73). A similar positive relation between FSH to central obesity in infertile females of reproductive age has also been reported (74).

FSH has also been independently associated with lean mass in 94 postmenopausal participants after adjustment for estrogen, testosterone, LH, parathyroid hormone, sex hormone binding globulin (SHBG) and urine N-telopeptide (75). The Study of Women Entering and in Endocrine Transition (SWEET) found significantly higher lean mass in premenopausal Sub-Saharan African females, as compared to postmenopausal females, with a negative correlation between FSH and lean mass (76).

However, several groups reported an inverse relationship between FSH levels and BMI in women, particularly those in the reproductive age (77–82). This phenomenon can be explained by feedback FSH inhibition by estrogens arising from adipose tissue. For example, a study from France reported that non-obese reproductive-age females undergoing infertility workups had higher levels of gonadotropins and estradiol compared to obese women (78). Another study found an inverse relationship between FSH and BMI in reproductive age females over 326 IVF cycles (77). Overweight/obese fertile women from Italy had lower FSH, LH, estradiol and inhibin B in the early follicular phase (79). The same scenario was reported in post- and perimenopausal females. For instance, Penn Ovarian Aging Study compared abdominal MR images and hormonal levels in women at different time points and demonstrated a positive relationship between estradiol and visceral fat, but a negative one was found between FSH and visceral fat (13). Furthermore, data from the 11-year follow-up SWAN study demonstrated that obesity is associated with low FSH trajectory in women of all ethnicities (80). According to The Pan-Asia Menopause (PAM) Study, gonadotropins and estradiol had a strong positive correlation with BMI. Interestingly, estrogen and LH levels were dependent on age, whereas FSH was not (81). Another study, conducted among 73 postmenopausal Serbian women, found higher FSH in normal weight individuals than in obese females (82).

These observations are consistent with those in girls, particularly among pubertal girls who underwent bioelectric impedance measures of body fat >29%. Sorensen and Juul demonstrated that girls within this cohort had significantly lower LH and FSH levels vs. normal weight comparators (83). Likewise, Bouvattier et al. observed a negative correlation between LH, FSH and GnRH responses regarding body mass index among perimenarchial and young adult girls (84).

No significant correlation between BMI and FSH was identified in observational studies of males regardless of age (85–88), except that one cross-sectional study reported that body mass index was negatively related to FSH, inhibin B, and testosterone levels in adult men (89). However, very recent data from a randomized clinical trial suggest that high serum FSH levels cause an increase in body fat in the absence of changes in other hormones. A two-arm open-label randomized clinical study included 58 men with prostate cancer, who were randomly assigned to orchiectomy or GnRH agonist treatment for 24 weeks (90). Notably, serum FSH levels increased after orchiectomy, while GnRH agonist injections inhibited FSH secretion (91). Men treated with orchiectomy experienced greater increases in total fat mass, subcutaneous adipose tissue mass, and weight at 48 weeks as compared to men treated with GnRH agonist (90). This is the first intervention study to demonstrate that FSH regulates body fat in human.

Limited data also suggests that serum FSH may be related to metabolic syndrome. For example, one cross-sectional study, of 320 Polish women reported FSH to be a better indicator of increased risk for metabolic syndrome than SHBG levels (92). Serum FSH also appeared to be more accurate in metabolic syndrome prediction compared with leptin or C-reactive peptide in menopausal females (93).

The role of FSH in non-alcoholic fatty liver disease (NAFLD) has not been well-established. However, a few studies have reported an association between serum FSH levels and fat deposition in the liver, detected by ultrasonography (94, 95). For example, the 2014 Survey on Prevalence in East China for Metabolic Diseases conducted among women over 55 years of age have revealed that serum FSH levels were negatively associated with NAFLD (94). In an adjusted model for waist circumference and HOMA-IR, FSH levels were not associated with mild hepatic steatosis, however the association of FSH with moderate-severe hepatic steatosis remained evident (*P* for trend <0.01) (94). Similarly, another cross-sectional study conducted among 71 elderly (i.e., 60 years of age or older) patients from China showed that the “normal” diurnal rhythm of FSH was independently associated with NAFLD (95).

### FSH Action on Body Fat in Mice

**Figure 1:**
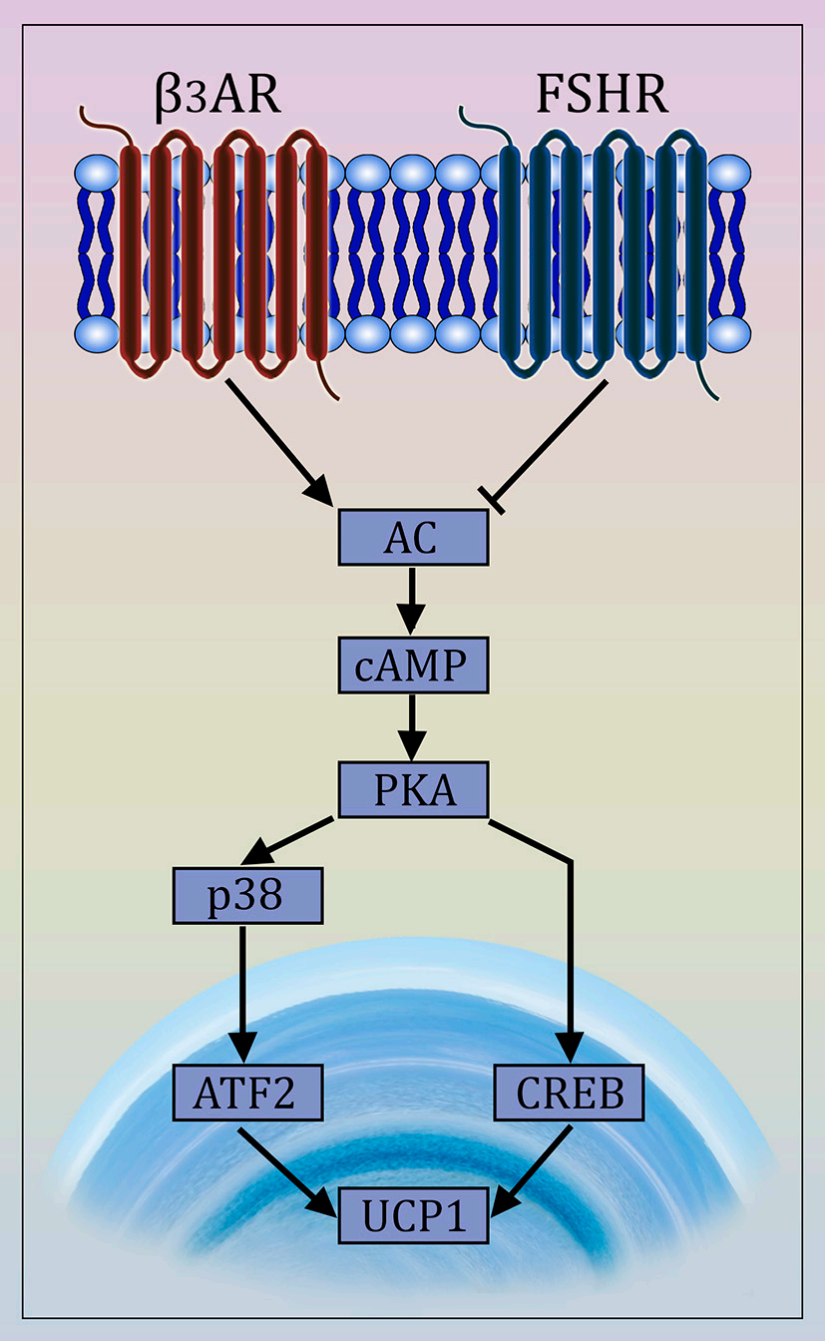
Mechanism of Action of FSH on Adipocytes. The newly described FSH signaling pathway opposes β_3_ adrenergic signaling. The latter is known to cause the transdifferentiation of white to beige adipocytes via interaction of the β_3_ receptor with a Gα_s_ protein that stimulates cAMP production and activates the MAP kinase p38 and the transcription factor ATF2, which then translocates to the nucleus causing the transcriptional activation of the *Ucp1* gene (98, 99). FSH opposes this action by interacting with a Gα_i_-coupled FSH receptor, also involving CREB-mediated pathway (9, 97).

There is compelling evidence for FSHR expression in chicken, murine and human adipocytes (9, 96, 97). FSH directly stimulates primary murine adipocytes and 3T3-L1 cells through Gα_i_-coupled FSHR (Figure 1), resulting in the up-regulation of core fat genes, such as *Fas, Lpl*, and *Pparg*, and the induction of lipid biosynthesis (9). Moreover, FSHR activation leads to cAMP reduction and subsequently UCP1 inactivation in ThermoMouse-derived differentiated brown fat cells (9).

We have fine-mapped the receptor-binding epitope of FSHβ and developed a blocking antibody capable of binding to this motif to prevent FSHβ/FSHR interaction (9, 10). Injection of this anti-FSHβ antibody in various murine models, including ovariectomized mice and mice either pair-fed on a high-fat diet or allowed ad libitum access to normal chow, caused a significant reduction in visceral, bone marrow and subcutaneous fat (9). The FSH antibody also significantly increased bone mass in ovariectomized mice (9, 70). These phenotypes were recapitulated in haploinsufficient *Fshr*^+/−^ mice, indicating a dominant role of FSH in bone and body fat regulation. Interestingly, anti-FSHβ antibody failed to decrease adiposity in *Fshr*^+/−^ male mice fed on a high-fat diet, proving FSH specificity (9). These observations were reproduced contemporaneously in other centers, using various laboratory methods (9, 100).

FSH blockade in mice also led to the up-regulation of brown adipocyte genes, such as *Cox7, Cox8a, Ucp1*, and *Cidea*, in visceral fat indicating “beiging,” which, we found, was occurring independently of sympathetic tone (9). However, it is not clear as to whether the beige adipocytes were the product of transdifferentiation of white adipocytes or if they were formed from a specific precursor (101, 102). Using *in vivo* fluorescence imaging on the IVIS platform, FSH blockade in ThermoMice triggered UCP1 transcription in brown-fat-rich areas initially with increases in white fat-predominant regions. The production of mitochondria-rich, thermogenic “beige” adipose tissue with the anti-FSHβ antibody was further substantiated using PhAM mice, and by documenting elevated basal energy expenditure in metabolic cage studies (9). Interestingly, FSH blockade did not affect glucose or insulin metabolism (92, 93).

Other studies have found a direct correlation between abdominal fat mass and *FSHR* mRNA expression in female chickens (96). FSH was found to alter lipid metabolism by affecting the expression of *Dci, Lpl, RarB, Rdh10, Dgat2*, and *Acsl3* genes, shifting fatty acid and retinol metabolism, and altering PPARγ signaling (96). Interestingly, FSH has also been shown to inhibit hepatic cholesterol metabolism. FSH was found to interact with FSHRs in HepG2 cells, reducing LDLR levels (103). Moreover, FSHR knockdown with specific siRNA in mice demonstrated lower LDLR (103), suggesting that FSH may be indirectly involved in the pathogenesis of NAFLD. Finally, in a Chinese cohort, rising FSH levels correlated positively with serum cholesterol and LDL levels in postmenopausal women (104).

### FSH Action on Cardiovascular System

Males receiving androgen deprivation therapy (ADT) for prostate cancer have an increased risk of cardiovascular dysfunction, atherosclerosis and thrombosis (105–107). For example, it has been shown recently that FSH promotes the development of cardiovascular risk in ADT-treated males (18). Moreover, several studies in females have demonstrated effects of FSH on cardiovascular risk measures, such as coronary artery calcium deposition and carotid intima-media thickness. For example, the SWAN study showed that in 856 women who never reported a stroke or a heart attack, FSH trajectory was correlated positively with intima-media thickness(17). Furthermore, a sub-study of the Prospective Army Coronary Calcium project, called the Assessment of the Transition of Hormonal Evaluation with Non-invasive Imaging of Atherosclerosis, showed that serum FSH levels were associated with the number of aortic plaques in 126 women undergoing menopausal transition using contrast-enhanced CT angiography and carotid ultrasound (16). However, a 22-site population-based Survey on Prevalence in East China for Metabolic Diseases and Risk Factors, showed a negative association between FSH levels and cardiovascular risk (108). The study had a cross-sectional design and any causal relationship between FSH levels and cardiovascular risk factor factors could not be established.

Mice receiving ADT have been used to study the relation of serum FSH and cardiovascular disease (CVD) development. The interaction of FSH with FSHR on monocytes has been shown to up-regulate RANK expression and promote monocytic infiltration of atherosclerotic plaques (18). T_h_1 helper cells then release RANKL, which activates RANK on monocytes, leading to osteoclast formation. Osteoclasts resorb calcified areas and provoke atherosclerotic plaque instability, increasing the risk of rupture and thrombosis (18). In a second study, serum FSH levels were also found to be significantly lower in mice treated with GnRH antagonists, as compared to animals getting GnRH agonist or orchiectomized (109). The first group displayed less fat mass, at least a two-fold lower atherosclerotic plaque burden, high levels of high-density lipoproteins (HDL), and reduced serum low-density lipoproteins (LDL) compared to the latter two groups. Although all animals developed fatty changes in the aortic wall, the necrotic regions were dramatically smaller in the first group (109). This suggests that increased CVD risk in ADT cannot be explained solely by hypoandrogenemia, and may relate to changes in serum FSH. Furthermore, it has also been surmised that, as atherosclerotic plaque development is dependent on neovascularization (110), FSH may act by stimulating new vessel formation [as effectively as vascular endothelial growth factor (111, 112)] via FSHR present on vascular endothelial cells (7). The mechanism includes the stimulation of VCAM-1 synthesis by FSHR expressed on endothelial cells. VCAM-1 then recruits monocytes to affect their migration and differentiation into macrophages that accumulate lipid droplets and eventually become foam cells (17, 113). Finally, FSH may elevate production of cytokines, namely IL-6 and TNFα, from macrophages to cause low-grade inflammation, atherosclerosis development and insulin resistance (114). We have documented this direct action in osteoclasts (115).

### FSH Action in Oncogenesis

FSH levels are elevated in ovarian cancer (116, 117). Furthermore, epithelial and endothelial FSHRs have been detected in various cancer types, including prostate (118, 119) and ovarian cancers (120–122), as well as in established cancer cell lines, namely prostate cancer cell lines DU145 (118) and PC-3 (118, 119), ovarian cancer cell lines including OVTOKO, CaOV-3, RNG1, OVCAR-3, and TOV-21G (121–126). Endothelial FSHR was detected by immunohistochemical and immunoblotting analysis in samples obtained from >1,000 patients with breast, prostate, colon, pancreas, urinary bladder, kidney, lung, liver, stomach, testis, and ovarian cancer (7). Recent data indicates that these FSHRs are signaling-efficient. In particular, endothelial FSHR expression is associated with vascular remodeling and tumor angiogenesis (6, 7), whereas epithelial FSHR induces cell proliferation (118–120), migration, and cancer cell invasion (127).

Interestingly, murine T-cells directed against FSHR- positive ovarian cancer cells showed increased survival without causing toxicity (122). FSHR stimulation upregulated Oct4 expression via the Erk1/2 pathway in epithelial ovarian cancer (128). Epithelial-to-mesenchymal transition in ovarian cancer was also stimulated through PI3K/Akt-Snail signaling (129). It has been suggested that FSH stimulates ovarian cancer cell proliferation via FSHR isoform 3, which is not coupled with G-proteins and not associated with cAMP production, but activates the Erk pathway in a Ca^2+^-dependent manner (130, 131).

Cancer cells express abundant receptors to various growth factors, suggesting the potential possibility of restricting cancer growth through antibody-mediated blockade of these receptors (132, 133). Unfortunately, the delivery of antibodies through the endothelium is poor and high doses are prone to cause toxicity (134, 135). To avoid this problem, a different approach, notably targeting the tumor vasculature, has been proposed. However, two major groups of extensively studied agents targeting tumor vessels have proven limitations and lack efficacy. Antiangiogenic agents reduce the action of various growth factors inside the tumor, preventing new blood vessel formation (136–139). Their maximum effect is tumor shrinkage and these agents have failed to improve survival (140, 141). The second group of agents, namely vascular disrupting agents, affect mature vessels, rearranging the endothelial cytoskeleton and increasing vascular wall permeability (142), thus disrupting blood supply and leading to extensive central necrosis of a tumor (143); this nonetheless leaves viable peripheral neoplastic tissue that subsequently repopulates the necrotic area (144–146). A new promising direction for anticancer target therapy is to cause peritumoral infarction using truncated tissue factor (tTF) coupled to ligands that are highly specific for FSHR (147). Antihuman FSHR antibody, conjugated with tTF, binds the FSHR, which is abundant in peritumoral endothelium, initiating blood clotting with subsequent blood supply disruption and tumor necrosis (148). Interestingly, the vasculature of bone and fat has not been shown to express FSHRs: thus, such therapy will most likely cause no issues (8, 149). However, their presence in the female reproductive system may limit anti-FSHR-tTF treatment. This approach still needs extensive investigation in the future and provokes extensive discussion on the development of a cancer therapies based on agents tethered to anti-FSHR antibodies (19).

## Conclusion

In the transitional phase of a women's reproductive life to menopause, the risk for osteoporosis, obesity and CVDs increase concurrently. Along with declining estrogen levels, sharply rising FSH levels have now been implicated in the pathogenesis of these diseases. It is now well-known that bone loss begins even before estrogen levels are altered in the perimenopause (150).

Several key findings have emerged relating serum FSH to bone loss, obesity, and perhaps even cardiovascular risk and cancer. First, it is clear that FSH directly impacts bone cells—osteoclasts and osteoblast precursors. The underlying mechanisms include a direct action on osteoclasts through the enhancement of RANKL signaling, and indirect actions to increase the expression of RANK in osteoclasts (151) and stimulate the synthesis of pro-resorptive cytokines, including TNFα, IL1β, and IL-6. Studies also conclusively demonstrate the expression of functional FSHRs on adipose tissue (97), which, when blocked by an FSH antibody, result in a profound reduction of body fat and generation of thermogenic “beige” adipose tissue (152). Together, the studies form the framework for using a humanized FSH antibody for the simultaneous treatment of two public health hazards—obesity and osteoporosis—with a single agent. Admittedly speculative at this stage, an increased risk of cardiovascular event(s) among postmenopausal women may also be in part attributable to subclinical atherosclerosis promoted by sharply rising FSH levels (153). Finally, certain cancers, prominently ovarian tumors in which oncogenic signaling through the FSHR can be proven may be amenable to novel FSH-based therapeutic agents.

Thus, positive correlations between rising FSH levels and a plethora of illnesses like obesity, osteoporosis, cardiovascular pathology, and cancer changes our view of FSH from monogamously associated with fertility to a much broader view of the role of this “gonadotropin” in other medical conditions and in human physiology. It is therefore now conceivable that we question whether FSH is a true aging hormone. By developing new treatment approaches that target this gonadotropin, we may in the future be able to treat multiple age-related diseases perhaps even with a single drug.

## Author Contributions

DL wrote the manuscript with support from S-MK, AR, IA, SJ, BA, CT, AK, and LS. TY, MZ, and RA helped supervise the project. All authors provided critical feedback and helped shape the manuscript.

### Conflict of Interest Statement

MZ is a named inventor on a patent related to FSH and bone, owned by Icahn School of Medicine at Mount Sinai. MZ will receive royalties and/or licensing fees per Mount Sinai policies, in the event the patent is commercialized. MZ also consults for Merck, Roche, and a number of financial consulting platforms. The remaining authors declare that the research was conducted in the absence of any commercial or financial relationships that could be construed as a potential conflict of interest.
